# Acupuncture for Essential Hypertension: A Meta-Analysis of Randomized Sham-Controlled Clinical Trials

**DOI:** 10.1155/2014/279478

**Published:** 2014-03-04

**Authors:** Dong-Ze Li, Yu Zhou, Yi-Ning Yang, Yi-Tong Ma, Xiao-Mei Li, Jing Yu, Yan Zhao, Hui Zhai, Lixing Lao

**Affiliations:** ^1^Department of Cardiology, The First Affiliated Hospital of Xinjiang Medical University, No. 137 Liyushan South Road, Urumqi, Xinjiang 830054, China; ^2^Department of Acupuncture-Moxibustion and Tuina, The First Affiliated Hospital of Xinjiang Medical University, No. 137 Liyushan South Road, Urumqi, Xinjiang 830054, China; ^3^Department of Cardiology, The Traditional Chinese Medicine Hospital of Luzhou Medical College, Sichuan, Luzhou 646000, China; ^4^Department of Information, The library of Xinjiang Medical University, No. 137 Liyushan South Road, Urumqi, Xinjiang 830054, China; ^5^School of Chinese Medicine, The University of Hong Kong, 10 Sassoon Road, Pokfulam, Hong Kong

## Abstract

*Background*. Acupuncture is frequently advocated as an adjunct treatment for essential hypertension. The aim of this review was to assess its adjunct effectiveness in treating hypertension. *Methods*. We searched PubMed, the Cochrane Library, EMBASE, and the Chinese databases Sino-Med, CNKI, WanFang, and VIP through November, 2012, for eligible randomized controlled trials that compared acupuncture with sham acupuncture. Outcome measures were changes in diastolic (DBP) and systolic blood pressure (SBP). *Results*. A total of 4 randomized controlled trials were included. We found no evidence of an improvement with the fact that acupuncture relative to sham acupuncture in SBP change (*n* = 386; mean difference = −3.80 mmHg, 95% CI = −10.03–2.44 mmHg; *I* ^2^ = 99%), and an insignificant improvement in DBP change (*n* = 386; mean difference = −2.82 mmHg, 95% CI = −5.22–(−0.43) mmHg; *I* ^2^ = 97%). In subgroup analyses, acupuncture significantly improved both SBP and DBP in patients taking antihypertensive medications. Only minor acupuncture-related adverse events were reported. *Conclusions*. Our results are consistent with acupuncture significantly lowers blood pressure in patients taking antihypertensive medications. We did not find that acupuncture without antihypertensive medications significantly improves blood pressure in those hypertensive patients.

## 1. Introduction

Essential hypertension is the most common cardiovascular disease (CVD), affecting about one billion individuals worldwide. The prevalence and incidence of hypertension tend to rise with age. Hypertension correlates closely with vascular morbidity and is a significant independent and well-characterized risk factor for other CVD, such as stroke and kidney disease. If the rise in blood pressure (BP) with age could be diminished, then the prevalence of hypertension, CVD, and cerebrovascular diseases could be greatly reduced. However, hypertension continues to be either untreated or uncontrolled in most individuals. Several classes of drugs can lower BP, but their availability, cost, and unwanted side effects have limited the effective control of hypertension to only about 50% of patients. Lifestyle interventions, such as exercise, weight loss, and salt intake restriction, can also lower BP, but these practices can be difficult to achieve and maintain. Therefore, there has been a growing interest in acupuncture as a treatment for hypertension.

Acupuncture is an ancient treatment technique anchored in traditional Chinese medicine (TCM) that has been used to treat symptoms related to hypertension for centuries [1]. Physicians and patients in China, South Korea, and Japan have considered it as an effective adjunctive treatment, while, in the west, its use has been increasing.

The efficacy of acupuncture for lowering BP was suggested by many published case reports and uncontrolled trials that have shown significant associated reductions [2]. However, other reports have not shown significant effects in comparison to control subjects [3]. Sham procedures for acupuncture now exist, which are inert and indistinguishable from the real treatment to allow blinding of the treatment allocation in treatment trials. These sham procedures include penetrating acupuncture on nonacupuncture points, superficial skin puncture on acupuncture points, and nonpenetration with sham needle devices on acupuncture points [4]. There have been meta-analysis of studies of the efficacy of acupuncture for hypertension but they were generally associated with conflicting results [5]. Review papers have also been published on effects of acupuncture on hypertension but some of these have included interventions other than acupuncture, and several have not been systematic reviews. We therefore conducted a meta-analysis of all currently available randomized sham-controlled trials of acupuncture for hypertension.

## 2. Materials and Methods

### 2.1. Search Strategy

A systematic search of the Cochrane Library, EMBASE, and PubMed was conducted without any language restriction. We also searched Chinese databases, including Sino-Med, Wanfang, CNKI, and VIP. Publications available from the inception of each database through November 2012 were reviewed to identify available randomized sham-controlled trials of acupuncture for hypertension. The following keywords were used in English digital databases: “Blood pressure,” “Hypertension,” “Acupuncture,” “Electroacupuncture,” and “Auricular acupuncture.” The following terms were used in the Chinese database searches: “ZHEN” (which means “Acupuncture”) and “Gao Xue Ya” (which means “Hypertension”). We also carefully scanned the references of relevant publications to identify further publications. When questions arose related to either the design or outcomes of trials, corresponding authors were contacted to confirm the information that we extracted from their trials or to clarify any ambiguity.

### 2.2. Inclusion Criteria

Inclusion criteria included the following: (1) randomized sham-controlled clinical trials; (2) patients were diagnosed with hypertension, according to a systolic blood pressure (SBP) ≥140 mmHg and/or a diastolic blood pressure (DBP) ≥90 mmHg, or use antihypertensive drugs; (3) patients in the experimental group were treated with acupuncture, electroacupuncture, or auricular acupuncture more than once either with or without antihypertensive drugs; (4) placebo (sham) procedures were used; (5) the study included an available clinical database.

### 2.3. Exclusion Criteria

Exclusion criteria included the following: (1) nonrandomized studies; (2) studies involving other forms of acupuncture, such as transcutaneous electrical nerve stimulation or laser acupuncture; (3) duplicate reporting with same results; (4) a lack of follow-up outcome data about BP; (5) if controls were given complementary or alternative therapies of which the efficacy is not yet established (e.g., herbal medicine).

### 2.4. Study Characteristics and Extraction

Following data were extracted independently by two of the authors (D. Li and Y. Zhou): (1) details of participants (e.g., gender, age, hypertension grade, and risk factors); (2) trial design, sample size, blinding, intervention procedures, withdrawals, and dropouts; (3) net changes in SBP and DBP and/or mean BP before and after acupuncture treatment as available. Any disagreements about either inclusions or analyses were resolved by consensus or arbitration by a third reviewer (Y. Yang). We contacted corresponding authors via e-mail to request further information when necessary.

### 2.5. Methodological Quality

The methodological quality of each included study was assessed by using the 5-point Jadad quality scale [6]. The Jadad scale focuses on three criteria: “randomization,” “double blinding,” and “withdrawals and dropouts” for assessing the quality of randomized controlled trial (RCT). RCTs were classified as high-quality if their Jadad score was ≥4 and low quality if their Jadad score was ≤3. Disagreements regarding methodological quality were resolved with discussion between reviewers.

### 2.6. Statistical Analysis

The meta-analysis and statistical analyses were performed by using Stata software v12.0 (Stata Corporation, College Station, TX, USA) and RevMan software v5.1 (The Cochrane Collaboration, Oxford, UK). In the absence of clinical heterogeneity, we synthesized the results in a meta-analysis and compared the mean BP change in outcome measures with baseline values to assess differences between the intervention and control groups. Weighted mean differences and 95% confidence intervals (CIs) were calculated. The mean effect size was calculated by using a random effects model as we assumed that each study assessed different acupuncture treatments and thus represented different effects. A fixed effect model was used when there was no significant heterogeneity [7]. Differences compared to sham controls were considered relevant. The variance of the change was inferred by using a correlation factor of 0.05 [8]. Heterogeneity was presented as significant when it was over 50% or *P* < 0.10. Publication bias was explored via a funnel-plot analysis. In case of heterogeneity, we attempted to identify and explain it by using subgroup analysis.

## 3. Results

### 3.1. Literature Search and Study Selection

**Figure 1:**
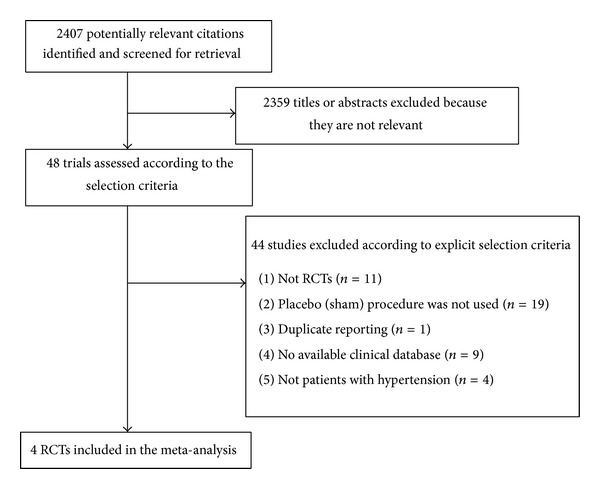
Flow diagram of study selection for the performed meta-analysis. RCT: randomized controlled trial.

An initial search of RCTs yielded 2407 potential literature citations. After screening titles and abstracts of all studies, 48 potentially relevant articles were selected and retrieved for a full-text assessment. Further screening for eligibility was performed by two independent reviewers by using inclusion and exclusion criteria. Finally, 4 RCTs were included, all being published in English (Figure 1).

### 3.2. Overall Study Characteristics

The 4 RCTs included a total of 386 patients with essential hypertension [3, 9–11]: 223 patients in the acupuncture group and 163 patients in the sham acupuncture group. The median BP at baseline was grade 1-2, and 44% of patients were taking antihypertensive medications. BP was measured at various time points (6th, 8th, and 10th weeks), with various methods, including 24 h ambulatory BP monitoring, mercury sphygmomanometer, and automated sphygmomanometer. Patients in only 2 studies took antihypertensive medications [9, 10]. The average follow-up period was 8 weeks (Table 1).

### 3.3. Acupuncture Treatment and Control Characteristics

Individualized acupuncture and/or standardized acupuncture were used 2-3 times a week for 6–10 weeks in the active acupuncture group in all 4 RCTs. All studies used sham acupuncture: 1 trial used superficial acupuncture in the control procedure; 2 used nonpenetrating acupuncture on either nonacupuncture points or real acupuncture points; and 1 used penetrating acupuncture on points irrelevant for lowering BP. No trials used sham electrostimulation on acupoints.

### 3.4. Methodological Quality

Studies were generally of good quality with a mean Jadad score of 4.75 (Table 1), of which 3 had a Jadad score of 5 [3, 9, 11]. One study received a Jadad score of 4 because it was not assessor-blinded [10]. All RCTs included in our meta-analysis were classified as high quality.

### 3.5. End Points

**Figure 2:**
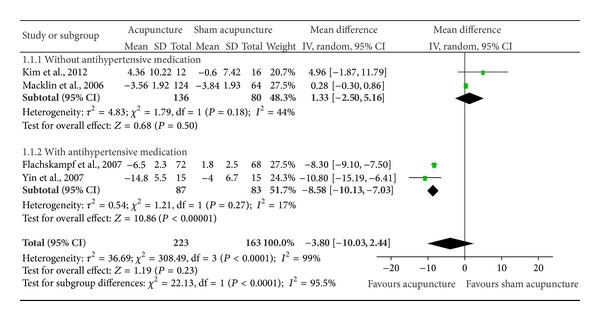
Pooled estimate of decrement in SBP with acupuncture treatment. SBP: systolic blood pressure.

**Figure 3:**
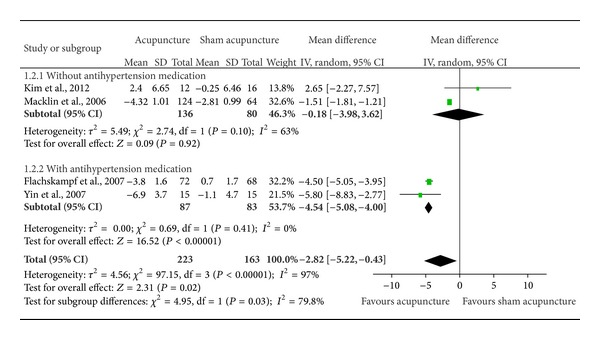
Pooled estimate of decrement in DBP with acupuncture treatment. DBP: diastolic blood pressure.

SBP and DBP changes between baseline and after acupuncture/sham interventions were reported in each of the 4 studies [3, 9–11]. No significant differences were found with the random-effects model between acupuncture and sham groups with respect to SBP change (*n* = 386; mean difference = −3.80 mmHg, 95% CI = −10.03–2.44 mmHg; *I* ^2^ = 99%) (Figure 2) or DBP (*n* = 386; mean difference = −2.82 mmHg, 95% CI = −5.22–(−0.43) mmHg; *I* ^2^ = 97%) (Figure 3).

Studies were significantly heterogeneous for SBP change (*P*_heterogeneity_ < 0.00001; *I* ^2^ = 99%) and for DBP change (*P*_heterogeneity_ < 0.00001; *I* ^2^ = 97%). In subgroup analyses, a significant benefit in both SBP (*n* = 170; mean difference = −8.58 mmHg, 95% CI = −10.13–(−7.03) mmHg; *I* ^2^ = 17%) (Figure 2) and DBP (*n* = 170; mean difference = −4.54 mmHg, 95% CI = −5.08–(−4.00) mmHg; *I* ^2^ = 0%) (Figure 3) was found for acupuncture among patients taking antihypertensive medications. For its part, among patients not taking antihypertensive medications, there was a significant improvement in DBP (*n* = 216; mean difference = −0.18 mmHg, 95% CI = −3.98–3.62 mmHg; *I* ^2^ = 63%) (Figure 3), but not in SBP (*n* = 216; mean difference = 1.33 mmHg, 95% CI = −2.50–5.16 mmHg; *I* ^2^ = 44%) (Figure 2).

### 3.6. Adverse Events

Four trials reported occurrences of adverse events. One study reported that 2 of their standardized acupuncture participants experienced hypertensive urgencies and 1 of their control participants experienced congestive heart failure [11]. Minor adverse events that occurred in the other 3 studies included pain and bleeding at the locus of needling [3, 9, 10].

## 4. Discussion

### 4.1. Overview of Findings

To the best of our knowledge, this is the first meta-analysis of acupuncture *versus* sham acupuncture for essential hypertension. In our review, we found that acupuncture according to TCM practices significantly lowered SBP and DBP in patients taking antihypertensive medications. For its part, acupuncture significantly lowered DBP, but not SBP, in patients who were not taking antihypertensive medications.

### 4.2. Mechanism of Acupuncture

In TCM, hypertension is conceptualized as being caused by emotional factors, constitutional weaknesses which render to individuals susceptible to disease, and poor diet and overexertion which lead to imbalances between yin and yang in the liver, spleen, and kidney. Mechanisms by which acupuncture are theorized to be therapeutic for hypertension according to Chinese medicine are by regulating yin and yang, reinforcing healthy qi, and expelling pathogenic factors [12]. Practitioners need to properly assess underlying causes of hypertension to apply appropriate acupuncture techniques [13]. The effectiveness of acupuncture depends upon the proper use of techniques that are difficult for physicians to master. These techniques include the angle and depth of needle insertion and the retention of the needle before withdrawal [14]. The use of different techniques by different practitioners can affect therapeutic outcomes.

For its part, according to Western medicine, therapeutic mechanisms of acupuncture are unclear, but some evidence suggests that acupuncture can affect the intrarenal renin-angiotensin system and sympathetic nervous and endocrine systems [15]. Acupuncture has been theorized to lower reflex-induced hypertension by modulating the activity of cardiovascular presympathetic neurons in the rostral ventrolateral medulla [16]. Some studies have shown acupuncture to inhibit the activation of neurons in the arcuate nucleus of the hypothalamus, ventrolateral periaqueductal gray nuclei in the midbrain, and nucleus raphe pallidus in the medulla, resulting in a reduced activity of premotor sympathetic neurons in the rostral ventrolateral medulla [17]. Acupuncture may also affect the endocrine system and lead to a decrease in plasma renin, aldosterone, angiotensin II, norepinephrine, and serotonin [18]. Acupuncture would represent a safe and effective adjunctive therapy for hypertension based upon both the TCM and Western medicine theories.

### 4.3. Comparisons with Other Studies

As early as the 1950s, results from many clinical studies have suggested beneficial effects of acupuncture for lowering BP in patients with essential hypertension [15]. In 1975, acupuncture was found to significantly reduce SBP and DBP in 24 out of 28 patients with essential hypertension [19]. Results from many studies in China have suggested that acupuncture was a good adjunctive therapy for treating hypertension [20], but the drawn conclusions were not credible because these studies were all either observational or case reports with small sample sizes, unrigorous designs, and control group interventions being medications or other BP lowering therapies. In recent years, 4 high-quality studies about acupuncture for lowering BP were published, 2 of which suggested that acupuncture lowered mean BP compared to sham acupuncture [9, 10], while the remaining 2 showed a no significant effect of acupuncture [3, 11]. Acupuncture has been shown to be a safe treatment for hypertension in most studies [20], and only minor adverse events from the treatment.

In our meta-analysis, we found that acupuncture was able to lower DBP only and to help antihypertensive medications to lower BP. Therefore, acupuncture alone would be unlikely to bring significant benefits to patients with essential hypertension. Results from many studies in China suggested that acupuncture is a safe and effective treatment for essential hypertension, lowering SBP (about 10–20 mmHg) and DBP (about 6–10 mmHg) with few adverse events [20]. Studies conducted in Western countries showed much smaller changes in SBP (−4.36–14.8 mmHg) and DBP (−2.4–6.8 mmHg) [9]. Therefore, our meta-analysis including Western and Korean studies may underestimate the effect of acupuncture for hypertension. Regional differences in risk and protective factors for hypertension and differences in acupuncture techniques could substantially affect the potential efficacy of acupuncture.

Our study provided evidence that acupuncture helped (lowered) BP in patients taking antihypertensive medications but had no effect for the patients without antihypertensive medications. According to the four RCTs [3, 9–11], Yin et al. [9] and Flachskampf et al. [10] used acupuncture as an adjunct to pharmaceutical management. At the same time, Flachskampf et al. [10] found that acupuncture had no effect on the thirty-five study subjects (19 receiving active, 16 sham treatment) who were not taking antihypertensive medication when enrolled in the trial, which was the same conclusion with our study. Beta-blockers, calcium antagonists, angiotensin-converting enzyme inhibitors/angiotensin receptor blockers, and diuretics are mainly used for the patients in the two papers [9, 10]. More than 1 antihypertensive agent was used in both the two trails [9, 10] for partial patients. Different kinds of antihypertensive medications were matching statistically between active acupuncture group and sham acupuncture group. The patients studied by Yin et al. [9] and Flachskampf et al. [10] had lower mean baseline BP (135/83 mm Hg, 131/81 mm Hg). Lastly, sham acupuncture at nonacupuncture points was used in the trials designed by Macklin et al. [11]; Kim et al. [3]; Flachskampf et al. [10], while noninvasive sham acupuncture was used in the trials designed by Yin et al. [9]. However, no evidence shows that they have an effect on the conclusions.

The duration and frequency of acupuncture treatment are not enough so that only acupuncture could not lower blood pressure. Chen et al.'s study [21] proved that short-term (one month) acupuncture did not decrease BP significantly and acupuncture may regulate the cardiovascular system through a complicated brain network from the cortical level, the hypothalamus, and the brainstem. Yin and Du [22] found that acupuncture (3 months) can decrease the immediate BP, lower more blood pressure with the time of acupuncture treatment going, and maintain the antihypertensive effect for primary hypertension. In the four papers [3, 9–11] of our meta-analysis, maybe the short duration (6–10 weeks) and the low frequency (2-3 times, a week) of only acupuncture treatment are the main reasons why only acupuncture for primary hypertension is inefficacious. Zhang et al. [23] found that acupuncture (5 times weekly, 12 weeks) had an effect on reducing BP, especially in synergy with medication. Antihypertensive medications could increase efficacy of acupuncture for hypertension. Therefore, without enough duration and frequency of acupuncture treatment in the four trials [3, 9–11], acupuncture is efficacious for patients with antihypertensive medications.

### 4.4. Limitations and Strengths of This Review

Although our review was thorough, we cannot be absolutely certain that all relevant RCTs were found. The number of studies included in our meta-analysis (4) was small with each study also having a relatively small sample size, which ranged from 30 to 192 patients. Only grades I and II hypertensive patients were included in studies, so conclusions regarding grade III hypertension could not be drawn from our results.

In future RCTs of acupuncture it would be beneficial if all acupuncture procedures were performed according to TCM theory, based upon diagnoses made by four diagnostic methods (inspection, auscultation, olfaction, and palpation), and performed according to syndrome differentiation.

## 5. Conclusions

Results from this meta-analysis of randomized sham-controlled trials provide evidence that acupuncture helped (lowered) BP in patients taking antihypertensive medications. Our results did not provide support that acupuncture alone significantly lowers BP in patients with hypertension. Larger RCTs with longer follow-up periods would help clarify the potential efficacy and safety of acupuncture for treating hypertension.

## Figures and Tables

**Table 1:** Characteristics of the RCTs selected for the meta-analysis.

Characteristics	Macklin et al. [11]	Kim et al. [3]	Flachskampf et al. [10]	Yin et al. [9]
Country	United States	Republic of Korea	Germany	Republic of Korea

Mean age (years) (Acu/Con)	57 (IND)-56 (STD)/53	52/52	59/58	52/54

Hypertension grades	1-2	1-2	1-2	1-2

Design	Prospective, double-blind, randomized, parallel group	Randomized, double-blind	Single-blind, randomized	Randomized, double-blind, placebo-controlled

Style	IND/STD	IND	IND	IND

Acupuncture treatment	IND (*n* = 64)/STD (*n* = 64) consisted of ≤12 generally twice a week 30 min acupuncture sessions provided over 6 to 8 weeks. Follow-up at 10 weeks, and 6 and 12 months	Acupuncture twice a week for 8 weeks. Follow-up at 8 weeks (*n* = 17)	Acupuncture 5 times weekly for first 2 weeks, and then 3 times weekly for following 5 weeks. Follow-up at 3 day, and 3 and 6 months (*n* = 72)	Acupuncture once every 3-4 days for 8 weeks. Follow-up at 4 weeks and 6 weeks (*n* = 15)

Sham acupuncture treatment	Invasive sham acupuncture (acupuncture at nonacupuncture points) (*n* = 64)	Sham acupuncture (acupuncture at nonacupuncture points superficially and bilaterally) (*n* = 16)	Sham acupuncture (acupuncture points without relevance for lowering BP) (*n* = 68)	Sham acupuncture (acupuncture superficially under the skin) (*n* = 15)

Outcome measures	BP at 10 weeks	24 h ambulatory BP at 8 weeks	(1) 24 h BP at 6 weeks (2) Daytime BP at 6 weeks (3) Nighttime BP at 6 weeks (4) Peak exercises (exercise at the maximal comparable workload) BP at 6 weeks	BP at 8 weeks

Antihypertensive medication	No	No	Yes	Yes

Jadad score	5	5	4	5

RCTs: randomized controlled trials; Acu: acupuncture group; Con: control group (sham acupuncture group); IND: individualized acupuncture; STD: standardized acupuncture; BP: blood pressure.
